# Quantifying biomarkers of axonal degeneration in early glaucoma to find the disc at risk

**DOI:** 10.1038/s41598-022-12036-4

**Published:** 2022-06-07

**Authors:** R. L. Bartlett, B. E. Frost, K. E. Mortlock, J. R. Fergusson, N. White, J. E. Morgan, R. V. North, J. Albon

**Affiliations:** 1 School of Optometry and Vision Sciences, Cardiff University, Cardiff, UK; 2 Cardiff Institute for Tissue Engineering and Repair, Cardiff University, Cardiff, UK; 3 Vivat Scientia Bioimaging Laboratories, Cardiff University, Cardiff, UK; 4 School of Biosciences, Cardiff University, Cardiff, UK

**Keywords:** Structural biology, Anatomy, Biomarkers, Diseases, Health occupations, Medical research, Pathogenesis, Risk factors, Signs and symptoms, Optics and photonics

## Abstract

To evaluate regional axonal-related parameters as a function of disease stage in primary open angle glaucoma (POAG) and visual field (VF) sensitivity. Spectral domain optical coherence tomography was used to acquire 20° scans of POAG (n = 117) or healthy control (n = 52) human optic nerve heads (ONHs). Region specific and mean nerve fibre layer (NFL) thicknesses, border NFL and peripapillary NFL, minimum rim width (MRW)/ area (MRA) and prelamina thickness; and volume were compared across POAG disease stages and with visual field sensitivity. Differences identified between early glaucoma (EG), preperimetric glaucoma (PG) and control (C) ONHs included thinner PG prelamina regions than in controls (*p* < 0.05). Mean border NFL was thinner in EG (*p* < 0.001) and PG (*p* = 0.049) compared to control eyes; and EG mean, and inferior and ST, border NFL was thinner than in PG (*p* < 0.01). Mean, superior and inferior PG peripapillary NFL were thinner than in controls (*p* < 0.05), and EG ST peripapillary NFL was thinner than in PG (*p* = 0.023). MRW differences included: PG SN and inferior less than in controls (*p* < 0.05); thinner EG mean regional, inferior, nasal, and ST MRW versus PG MRW (*p* < 0.05). Regional border NFL, peripapillary NFL, MRW, MRA, prelamina thickness (except centre, *p* = 0.127) and prelamina volume (*p* < 0.05) were significantly associated with VF mean deviation (MD). Novel axon-derived indices hold potential as biomarkers to detect early glaucoma and identify ONHs at risk.

## Introduction

Glaucoma remains the leading cause of irreversible blindness worldwide^1,2^ and it is estimated that over 70 million people are affected by the disease^3–5^. Intraocular pressure (IOP) is considered the major risk factor for primary open angle glaucoma (POAG)^6–8^, and current POAG treatment methods are aimed at lowering IOP via medical or surgical techniques^9–11^.

Irreversible vision loss in glaucoma is caused by the degeneration and/or loss of retinal ganglion cells (RGCs) and their axons^12,13^. RGC neuronal cell bodies are located within the ganglion cell layer, and their axons form the innermost retinal layer, the retinal nerve fibre layer (RNFL)^14,15^. Signs of glaucomatous optic neuropathy include characteristic changes in the appearance of the optic disc including enlargement of the optic cup and loss of the neuroretinal rim^16–19^ and RNFL defects due to loss of RGC axons^20–22^.

It is estimated that between 25 to 35% of RGCs and their axons are lost before irreversible visual field (VF) defects are detected using standard visual field tests^23,24^. Examination of the ONH and RNFL currently form the basis of glaucoma detection^14,15,21^. However, inconsistencies exist between clinicians in the subjective evaluation of the ONH to identify glaucomatous structural damage or disease progression^25–28^. It is likely that subtle glaucomatous disc changes are difficult to detect, identifying a need for novel optic disc assessment protocols in glaucoma. Since the introduction of optical coherence tomography (OCT)^29,30^ to the clinic setting, this technology has been used to quantify various RGC axon and disc related parameters to assess in vivo changes in glaucoma^31–34^.

RNFL measures have been reported to better detect progressive glaucoma disease, with inferior RNFL a better discriminator between healthy eyes and eyes with glaucomatous visual field loss than macula retinal thickness measures^35,36^. Furthermore, Lisboa et al.^37^ reported that RNFL measurements quantified by SD-OCT performed better than ONH and macula measures for detecting preperimetric glaucomatous damage^38^. Additionally, NFL thickness has been reported to provide a stronger correlation with visual field loss than optic disc cupping and neuroretinal rim area^39^. Jonas et al.^40^ reported sectorial neuroretinal rim loss, with pronounced inferior-temporal rim loss in eyes with modest glaucomatous damage (defined according to visual field Mean Deviation [MD]) and decreased superior-temporal rim width in eyes with moderate glaucoma. These studies support the use of regional RNFL and neuroretinal rim thickness measures as important clinical factors in glaucoma diagnosis and follow-up.

However, the use of RNFL thickness in glaucoma assessment does not provide sufficient information to stage glaucoma-related changes within the ONH^41^. Characterisation of changes to ONH structure such as prelamina cupping and lamina cribrosa alterations has been reviewed and proposed to enable more precise detection of early axonal loss in glaucoma^42,43^. Furthermore, peripapillary RNFL thickness measures in glaucoma disease do not take into consideration artefactual changes in RNFL that can arise owing to the angular relationship between RGC axons and the Bruch’s membrane opening (BMO) plane. To address this, Povazay et al.^44^ proposed neuroretinal rim measurements such as OCT-based minimum distance mapping to provide an optical correlate of true RNFL thickness around the ONH region.

Later, minimum rim width (MRW) and area (MRA) were reported to enhance glaucoma detection^45,46^, and correlate with RNFL and visual field loss^47^. However, Amini et al.^48^ reported that peripapillary NFL in glaucoma provided a stronger correlation with visual field sensitivity than MRW measures.

The optimal axonal parameter representative of RGC axon integrity to characterise glaucoma disease stage or be used in early detection of glaucomatous optic neuropathy remains undecided. Using high resolution OCT, this study aims to use novel indices of RGC axon integrity, including border NFL thickness (height above Bruch’s membrane termination) and prelamina volume; as well as regional analyses of other known indices; peripapillary NFL, MRW, MRA, and prelamina thickness, to characterise glaucomatous optic neuropathy as a function of POAG disease stage in attempt to determine an optimal index, or group index, for early detection of optic discs at risk of progressive damage.

## Methods

### Participant recruitment and demographics

This cross-sectional study included 59 POAG participants (mean age ± SD: 70.19 ± 8.43 years) and 26 control participants (mean age ± SD: 67.27 ± 5.06 years). Participants were recruited from the University Hospital of Wales, Cardiff and/or Cardiff University School of Optometry and Vision Sciences (CUS Optom). This study was approved by Wales Research Ethics Committee and CUS Optom ethics audit committee; investigations were carried out in accordance with the tenets and declaration of Helsinki. Participant demographics are presented in Table 1.

**Table 1:** Participant demographics for glaucoma participants and age-matched controls.

Characteristic	Control	PG	EG	MAG
	N = 52 eyes	N = 31 eyes	N = 63 eyes	N = 23 eyes
	Mean ± Standard Deviation			
Age (years)	67.27 ± 5.06	67.58 ± 9.09	71.16 ± 7.80	72.61 ± 7.76
Gender	26 F & 26 M	16 F & 15 M	32 F & 31 M	11 F & 12 M
MS (D)	1.10 ± 1.65	− 0.13 ± 2.85	0.20 ± 2.52	0.07 ± 2.26
VA (logMAR)	− 0.02 ± 0.09	0.05 ± 0.10	0.11 ± 0.13	0.15 ± 0.18
IOP (mmHg)	14.98 ± 3.31	13.39 ± 2.12*	13.36 ± 2.36*	12.13 ± 2.59*
AEL (mm)	23.69 ± 0.94	23.94 ± 1.72	23.90 ± 1.34	24.06 ± 1.35
CCT (μm)	560.60 ± 41.29	529.48 ± 31.14	534.14 ± 44.28	526.09 ± 32.60
ACD (mm)	2.84 ± 0.60	2.93 ± 0.68	3.14 ± 0.89	3.16 ± 0.87
VF MD (dB)	− 0.44 ± 1.21	− 0.25 ± 1.04	− 3.05 ± 1.65	− 11.19 ± 4.74

MS = mean sphere, VA = visual acuity, IOP = intraocular pressure, AEL = axial eye length, CCT = central corneal thickness, ACD = anterior chamber depth, VF MD = visual field Mean Deviation. *Note that POAG IOP represents treated IOP.

All participants underwent assessment of both eyes including best-corrected visual acuity, Goldmann applanation tonometry, slit-lamp biomicroscopy, and dilated stereoscopic examination of the ONH and fundus. Axial eye length was determined using an IOL-Master (Carl Zeiss, Meditec Inc, Germany). Central corneal thickness (CCT) and anterior chamber depth were measured with a Pentacam (Oculus Optikgeräte, Germany). Visual field status was determined using Humphrey Visual Field Analyser 24–2 SITA standard (Carl Zeiss Meditec Inc, Germany).

Participant inclusion criteria included a mean spherical correction within ± 6.00 dioptres, and less than 3.00 cylindrical correction. Control participants were required to have best-corrected visual acuity of 0.2 logMAR or better, intraocular pressure (IOP) ≤ 21 mmHg, normal visual fields, a normal appearance to the optic disc, and no ocular or systemic conditions affecting the ONH. Eyes with significant cataract or ocular conditions other than glaucoma that could affect the ONH structure or visual fields were excluded. Diagnosis of POAG by a Consultant Ophthalmologist was based upon typical glaucomatous optic disc features including diffuse or focal thinning/notching of the NRR^16,49^, IOP > 21 mmHg prior to topical treatment, and characteristic visual field defects^50,51^. POAG participants that displayed characteristic ON changes without visual field defects were classified as preperimetric glaucoma (PG). Participants were divided into three POAG groups according to visual field mean deviation: PG (no visual field defect); early glaucoma (EG; visual field mean deviation better than − 6 dB); and moderate-advanced glaucoma (MAG; visual field mean deviation worse than − 6 dB)^52,53^. Participants were excluded from the study if they displayed unreliable visual field testing, defined as fixation losses over 20% or false positive/negative errors over 20%^54,55^.

### Optical coherence tomography

Spectral domain optical coherence tomography (SD-OCT) scans (20° scan angle) centred on the ONH, comprising 512 × 512 × 1024 pixels, were acquired in both eyes of each participant using long wavelength SD-OCT. The latter was a custom-built research OCT device, with a superluminescent diode light source (1 micron ASE module, NP-Photonics, Tuscan, US) with a centre wavelength of 1040 nm (bandwidth 70 nm), fibre-optically connected to a 20:80 beam-splitter serving the sample and reference arm respectively^56^. The system used a grating-based spectrometer with a Goodrich SUI-LDH-1.7 camera and operated at 47,000 a-scans per second with an axial resolution of ~ 7 µm and transverse resolution of ~ 15 µm^57–59^. Prior to OCT image acquisition, the power of the OCT system was recorded to ensure it was less than 2.5mW at the cornea, below the maximum power limit for a 10-s duration exposure^60,61^.

### OCT image processing

Acquired spectral data in FD1 file format were converted to 16-bit TIFF image format using custom-written software, OCT1_FD1_v2.2 (MATLAB 2014b, Math-works, US). OCT tomograms were then aligned within the 3D image datasets using stack registration using the Fiji ImageJ (version 1.52a, National Institutes of Health, USA); Schindelin et al.^62^ plugin StackReg^63^. OCT image noise was reduced using a 3D median filter. Image brightness and contrast were adjusted to redistribute pixel intensities for optimal visualisation of the ONH. Image pixel to distance calibration was performed, accounting for the effect of axial eye length and tissue refractive index^64,65^, as described by Terry et al.^59^.

### Measurements of axonal-related parameters

**Figure 1:**
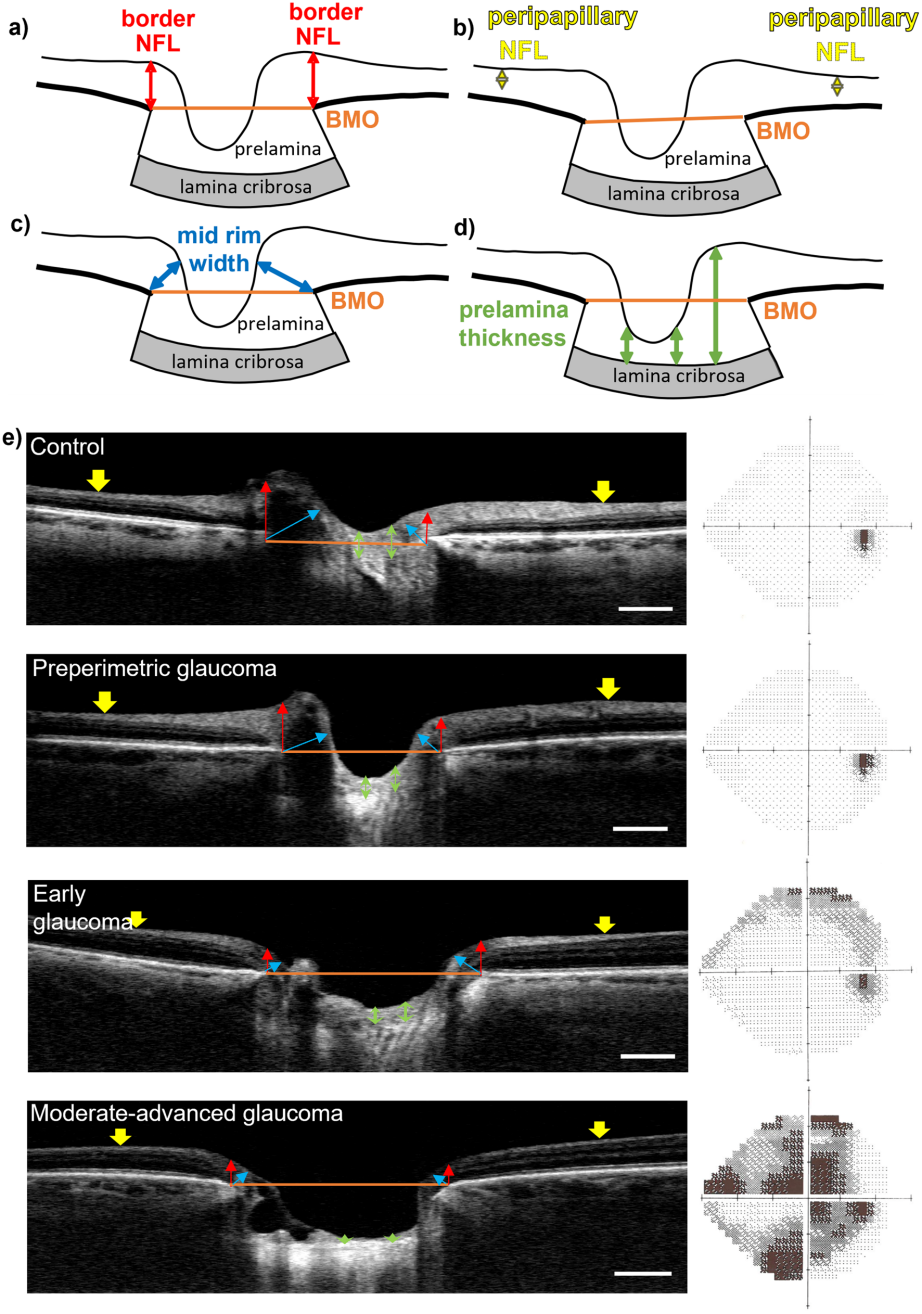
(**a**) Schematic optic nerve head (ONH) diagram depicting (**a**) measurement of border nerve fibre layer (bNFL); (**b**) peripapillary nerve fibre layer (pNFL) measured 1.7 mm from ONH centre; (**c**) minimum rim width (MRW); (**d**) prelamina thickness calculated as the difference between anterior lamina cribrosa (LC) and prelamina surface depths relative to Bruch’s membrane opening (BMO). (**e**) Representative OCT tomograms (inferior-nasal to superior-temporal axis) of optic nerve heads in Control, Preperimetric, Early Glaucoma and Moderate-advanced Glaucoma groups, with corresponding visual field plots. Axon-related parameters: border NFL (red arrows), position of peripapillary NFL measurement (yellow arrow heads), Bruch’s membrane opening (orange line), MRW (blue arrows), prelamina thickness (green arrows). Scale bar represents 500 µm. Visual field plots are normal for control and preperimetric groups; note that the black region on right hand-side represents the physiological blind spot. Increasing visual field loss is indicated, from early glaucoma to moderate-advanced groups, by the increased areas of grey and black.

Diagrams of optic nerve heads, indicating the position of axonal parameter measurements can be seen in Fig. 1a–d. The latter can also be observed in representative OCT tomograms of optic nerve heads within the groups: control, preperimetric glaucoma, early glaucoma and moderate-advanced glaucoma, with corresponding visual field plots in Fig. 1e.

#### NFL and MRW

Each OCT dataset was sliced radially at 45° intervals around the ONH centre to produce four OCT tomograms through the 3D ONH image datasets, with orientations: superior – inferior, nasal – temporal, and superior nasal – inferior temporal, and superior temporal – inferior nasal, enabling measurement of 8 regions. Within each OCT tomogram, Bruch’s membrane terminations were identified and used to create a reference plane across Bruch’s membrane opening (BMO) for subsequent measurements.

A novel measure, NFL thickness at the ONH border (border NFL) was measured as the vertical distance from each Bruch’s membrane termination to the NFL surface (Fig. 1a). Peripapillary NFL was measured 1.7 mm from the BMO centre in each tomogram (Fig. 1b). Minimum rim width (MRW) was defined as the minimum distance from each Bruch’s membrane termination to the inner limiting membrane as described by Povazay et al.^44^, as shown in Fig. 1c. Minimum rim area (MRA) was calculated, using MRW values, as described by Gardiner et al.^47^.

#### Prelamina thickness and volume

Regional (superior, inferior, nasal, temporal, superior temporal, inferior temporal, superior temporal, inferior nasal and centre) prelamina thickness was measured within each ONH OCT dataset. The central region was measured at the midpoint of the BMO reference plane within each OCT tomogram, with other regions being measured at the mid-points between BMO centre and BMO terminations. Prelamina thickness was calculated by subtracting prelamina surface depth from anterior lamina cribrosa surface depth, both measured axially with respect to BMO reference plane (Fig. 1d).

Prelamina volume of each 3D ONH OCT image dataset was quantified using Amira software (version 6.0, Thermo Fisher Scientific, UK). First a reference BMO plane area was created by generate a 2D surface from landmarks placed around the ONH border. Then, using a similar a protocol with the addition of the anterior prelamina, 3D volumetric surface was generated to quantify the optic cup volume. Next, this was repeated using the anterior lamina cribrosa surface, and the optic cup volume was subtracted from this volume to calculate prelamina volume.

### Statistical analysis

To overcome inter-eye correlation with the fellow eye, a common approach in ophthalmological research is either to average the data from fellow eyes, or to collect data from one eye only^66^. However, Murdoch et al.^67,68^ have suggested that it is ‘a waste’ to discard fellow eye data, and that appropriate statistical techniques, such as general linear mixed-effects models, can be applied to avoid data autocorrelation or having to discard data from the fellow eye^68,69^. Incorporating data from both eyes of a participant has the advantages of enhanced statistical power, more interpretable regression coefficients, greater precision of estimation and less sensitivity to missing data^66,68,70^.

In all statistical analyses performed in this study, data of each parameter (specific region or mean), acquired from both eyes (where possible) of each participant were used. To account for the inter-eye correlation of data between eyes of the same participant, general linear mixed-effects statistical models were constructed including a repeated measures component. The ‘lme4’ package was used to fit linear mixed-effects regression models within RStudio, version 1.2.1335 (http://cran.r-project.org/package=lme4). For each parameter measured e.g., superior border NFL, a linear mixed-effects regression model was developed. To optimise each model, a stepwise deletion of fixed effects (namely participant age, stage of glaucoma, axial eye length, anterior chamber depth, refractive error, central corneal thickness and intraocular pressure) was performed to determine the association between each fixed effect and the ONH parameter. Only significantly associated variables (*p* < 0.05) were included in regression models to account for effects on the axonal-related parameter. Inter-group differences (i.e., between the different stages of glaucoma) for each parameter were determined using Tukey post-hoc pairwise comparisons using ‘emmeans’ (http://cran.r- project.org/package = emmeans). Statistical differences were determined at *p* < 0.05.

Data normality was determined using histograms and the Shapiro–Wilk test with significance assumed at *p* < 0.05. Associations between each parameter and visual field mean deviation were determined using Pearson’s correlation coefficient.

### Ethics approval

Approved by South-East Wales Research Ethics Committee Panel C; reference number: 10/WSE03/24 and Cardiff University School of Optometry and Vision Sciences ethical committee; project number: 1299.

### Consent to participate

Informed written consent granted prior to participation.

### Consent for publication

None.

## Results

### Axon-related parameters as a function of glaucoma disease stage and visual field sensitivity

#### Border NFL

**Figure 2:**
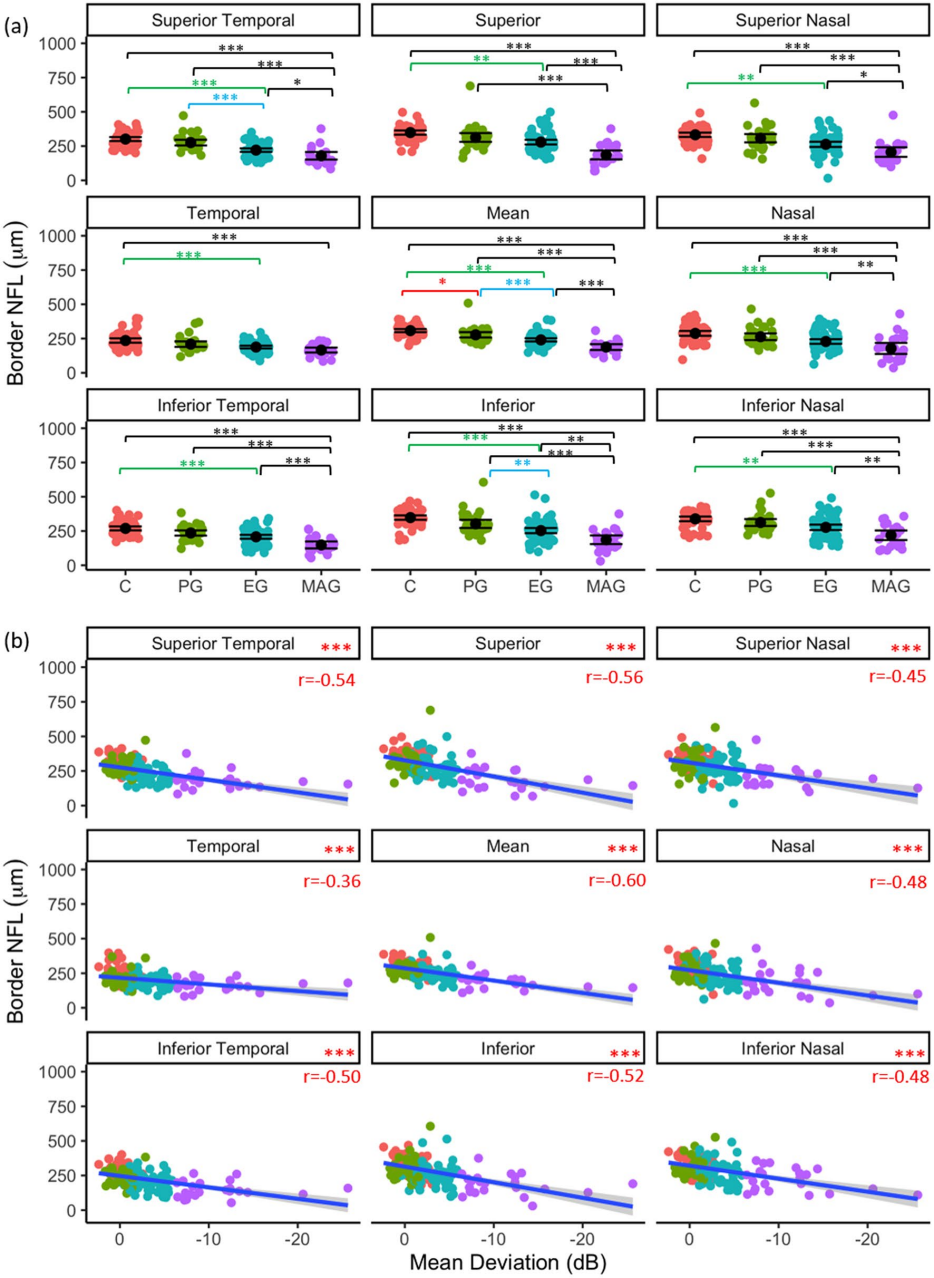
Regional border NFL as a function of glaucoma disease stage (**a**); black point represents mean and error bars indicate 95% confidence intervals, and (**b**) visual field status (VF MD). Blue line represents regression line and grey shading indicates 95% confidence intervals. Red text indicates significant Pearson’s correlation (r) values at *p* < 0.05. *p < 0.05, **p < 0.01, ***p < 0.001.

Regional border NFL inter-glaucoma stage differences and associations with visual field mean deviation are shown in Fig. 2a and b, respectively. Mean border NFL in the PG group was significantly less than in control eyes (*p* = 0.049). Compared to PG ONHs, average (*p* < 0.001), inferior (*p* = 0.007), and superior temporal (*p* < 0.001) border NFL were significantly less in EG ONHs. Border NFL was significantly less in MAG than in EG ONHs in all regions (*p* < 0.05), except temporal (*p* = 0.493). In all regions analysed, border NFL was significantly negatively correlated with VF MD (*p* < 0.001, Fig. 2b).

#### Peripapillary NFL

**Figure 3:**
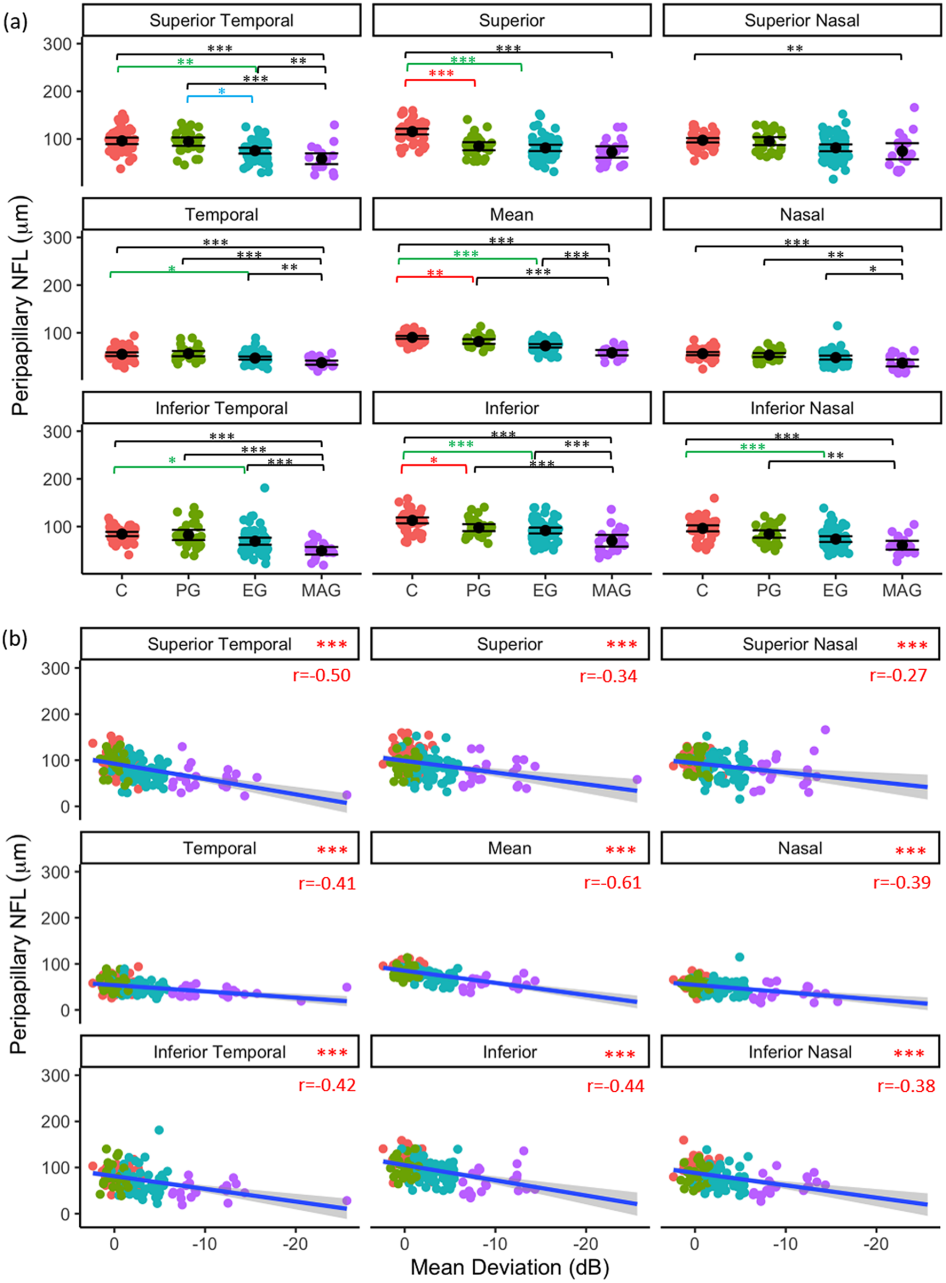
Regional pNFL as a function of glaucoma disease stage (**a**); black point represents mean and error bars indicate 95% confidence intervals, and (**b**) visual field status (VF MD). Blue line represents regression line and grey shading indicates 95% confidence intervals. Red text indicates significant Pearson’s correlation (r) values at *p* < 0.05. *p < 0.05, **p < 0.01, ***p < 0.001.

Regional peripapillary NFL inter-glaucoma stage differences and associations with VF MD are shown in Fig. 3a and b, respectively. Mean (*p* = 0.002), superior (*p* < 0.001), and inferior (*p* = 0.033) peripapillary NFL were significantly thinner in PG ONHs than in control eyes, and EG superior temporal peripapillary NFL was thinner than in PG ONHs (*p* = 0.023). MAG peripapillary NFL was thinner than EG in all regions (*p* < 0.05), except for superior (*p* = 0.674), superior nasal (*p* = 0.443), and inferior nasal (*p* = 0.135) peripapillary NFL. Peripapillary NFL was significantly negatively associated with VF MD in all regions (*p* < 0.001, Fig. 3b).

#### Minimum rim width (MRW)

**Figure 4:**
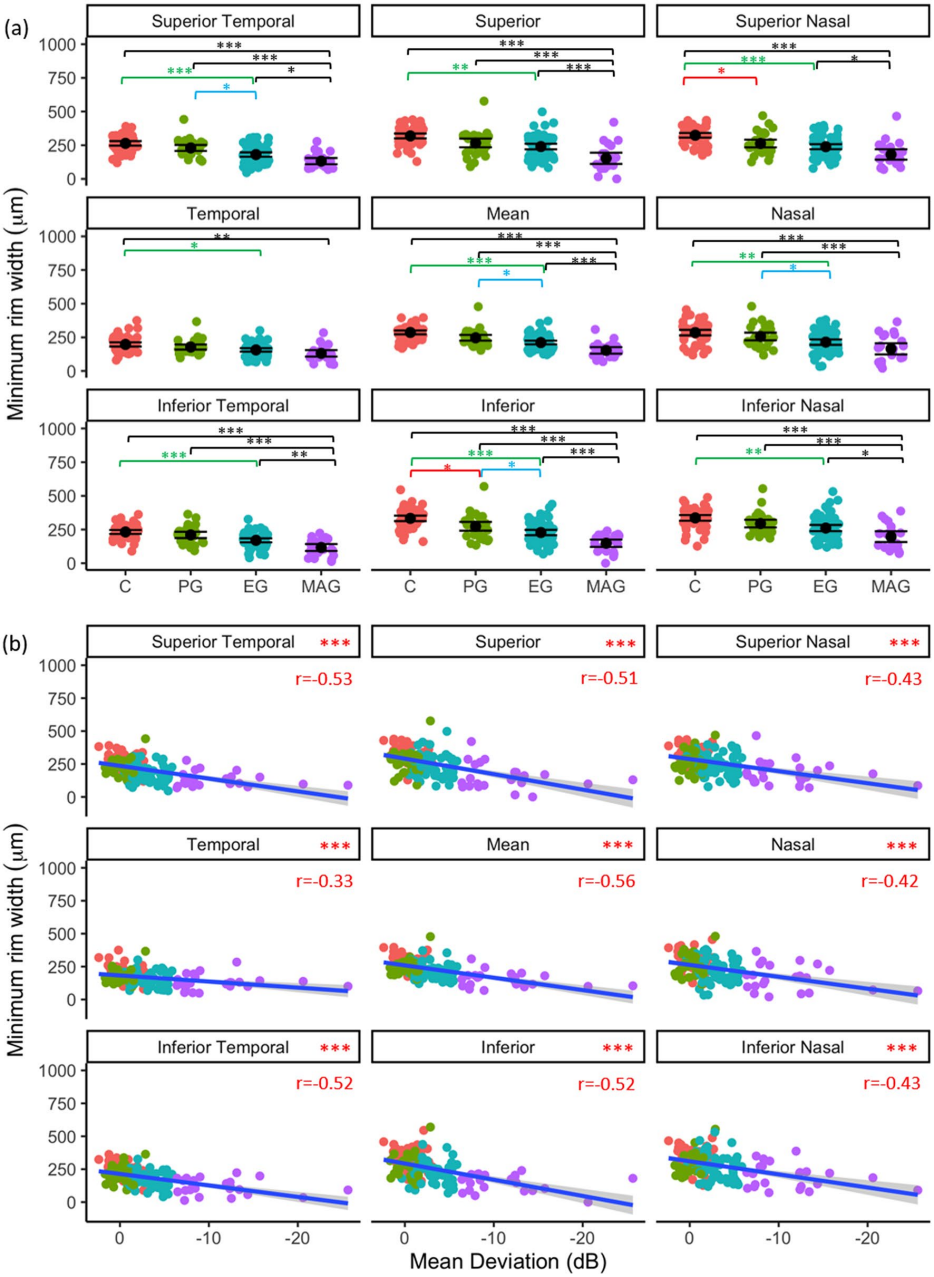
Regional MRW as a function of glaucoma disease stage (**a**); black point represents mean and error bars indicate 95% confidence intervals, and (**b**) visual field status (VF MD). Blue line represents regression line and grey shading indicates 95% confidence intervals. Red text indicates significant Pearson’s correlation (r) values at *p* < 0.05. *p < 0.05, **p < 0.01, ***p < 0.001.

Regional MRW inter-glaucoma stage differences and associations with VF MD are shown in Fig. 4a and b, respectively. MRW was significantly thinner in PG than controls in the inferior (*p* = 0.027) and superior nasal (*p* = 0.037) regions. PG MRW was greater than in EG group in the inferior (*p* = 0.042), nasal (*p* = 0.014) and superior temporal (*p* = 0.036) regions, including the regional mean (*p* = 0.014, Fig. 4a). MRW was less in MAG, compared to EG in all regions (*p* < 0.05), except nasal (*p* = 0.208) and temporal (*p* = 0.547). MRW was significantly negatively associated with VF MD in all regions (*p* < 0.001, Fig. 4b).

#### Minimum rim area (MRA)

**Figure 5:**
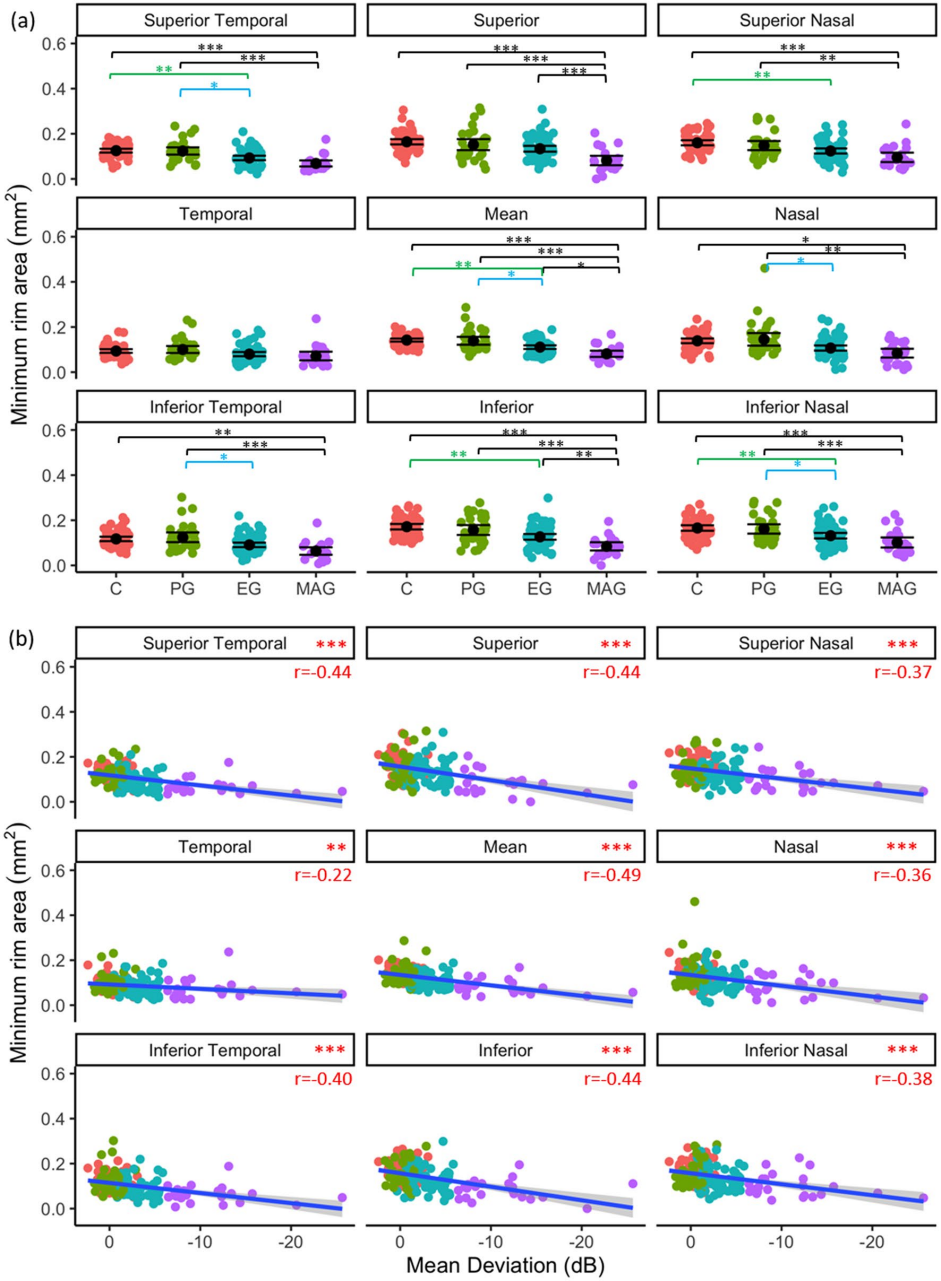
Regional MRA as a function of glaucoma disease stage (**a**); black point represents mean and error bars indicate 95% confidence intervals, and (**b**) visual field status (VF MD). Blue line represents regression line and grey shading indicates 95% confidence intervals. Red text indicates significant Pearson’s correlation (r) values at *p* < 0.05. *p < 0.05, **p < 0.01, ***p < 0.001.

MRA did not significantly differ between PG and control eyes in any region (*p* > 0.300). Mean MRA (*p* = 0.011), and MRAs in nasal (*p* = 0.020), inferior temporal (*p* = 0.036), superior temporal (*p* = 0.026), and inferior nasal (*p* = 0.031) regions were significantly less in EG compared to PG. Mean MRA (*p* = 0.012), and superior (*p* < 0.001), and inferior (*p* = 0.012) MRA, were significantly less in MAG, compared to EG ONHs (Fig. 5a). MRA regions and mean MRA were significantly negatively correlated with VF MD (*p* < 0.01, Fig. 5b).

#### Prelamina thickness

**Figure 6:**
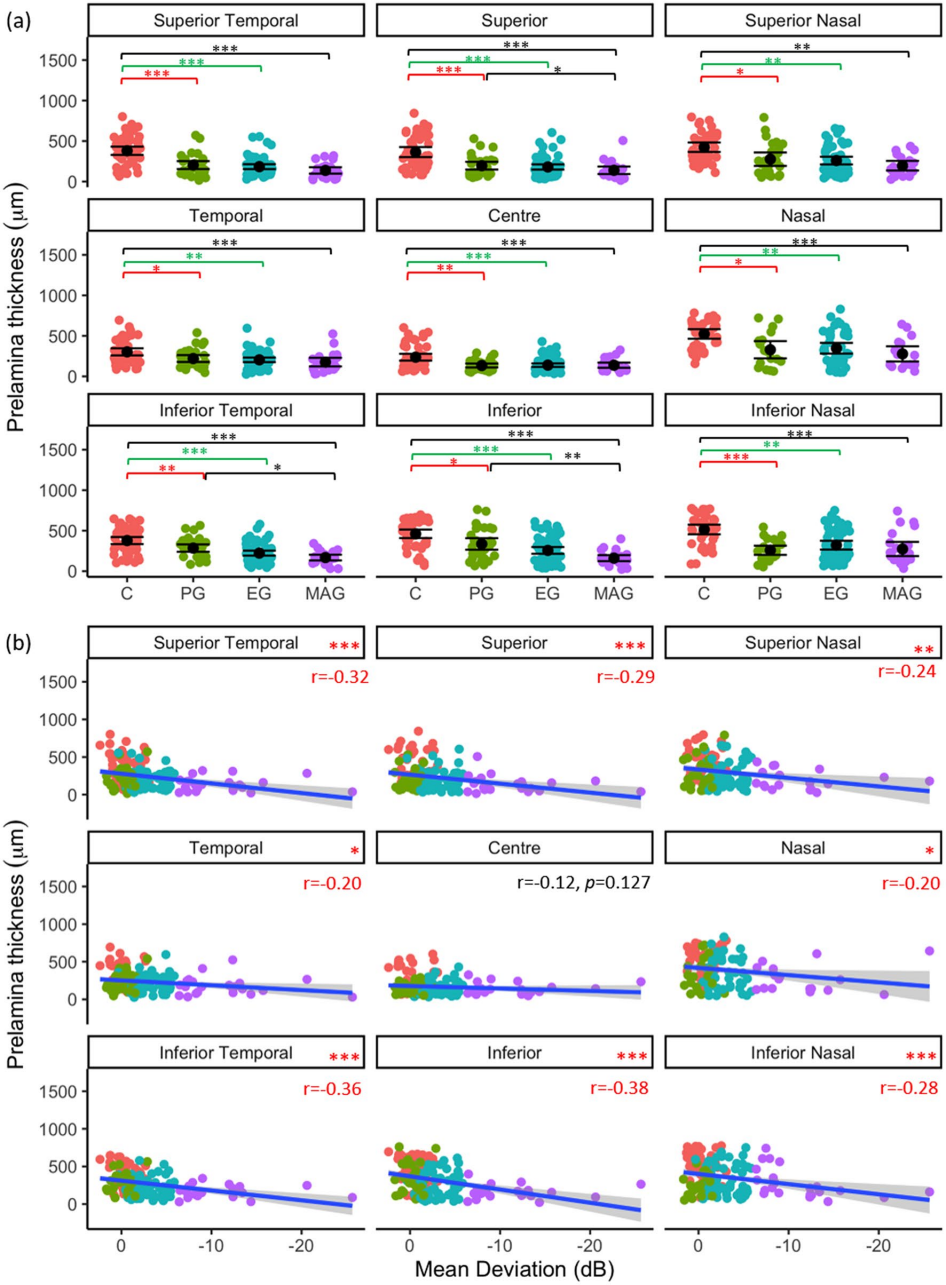
Regional prelamina thickness as a function of glaucoma disease stage (**a**); black point represents mean and error bars indicate 95% confidence intervals, and (**b**) visual field status (VFMD). Blue line represents regression line and grey shading indicates 95% confidence intervals. Red text indicates significant Pearson’s correlation (r) values at *p* < 0.05. *p < 0.05, **p < 0.01, ***p < 0.001.

The prelamina was thinner in PG than control eyes in all ONH regions (*p* < 0.05). No differences were identified between PG and EG (*p* > 0.20), or between MAG and EG prelamina thicknesses in any region (*p* > 0.07, Fig. 6a). A significant negative correlation between VF MD and prelamina thickness in all ONH regions (except centre (*p* = 0.127, Fig. 6b) was found (*p* < 0.05).

#### Prelamina volume

**Figure 7:**
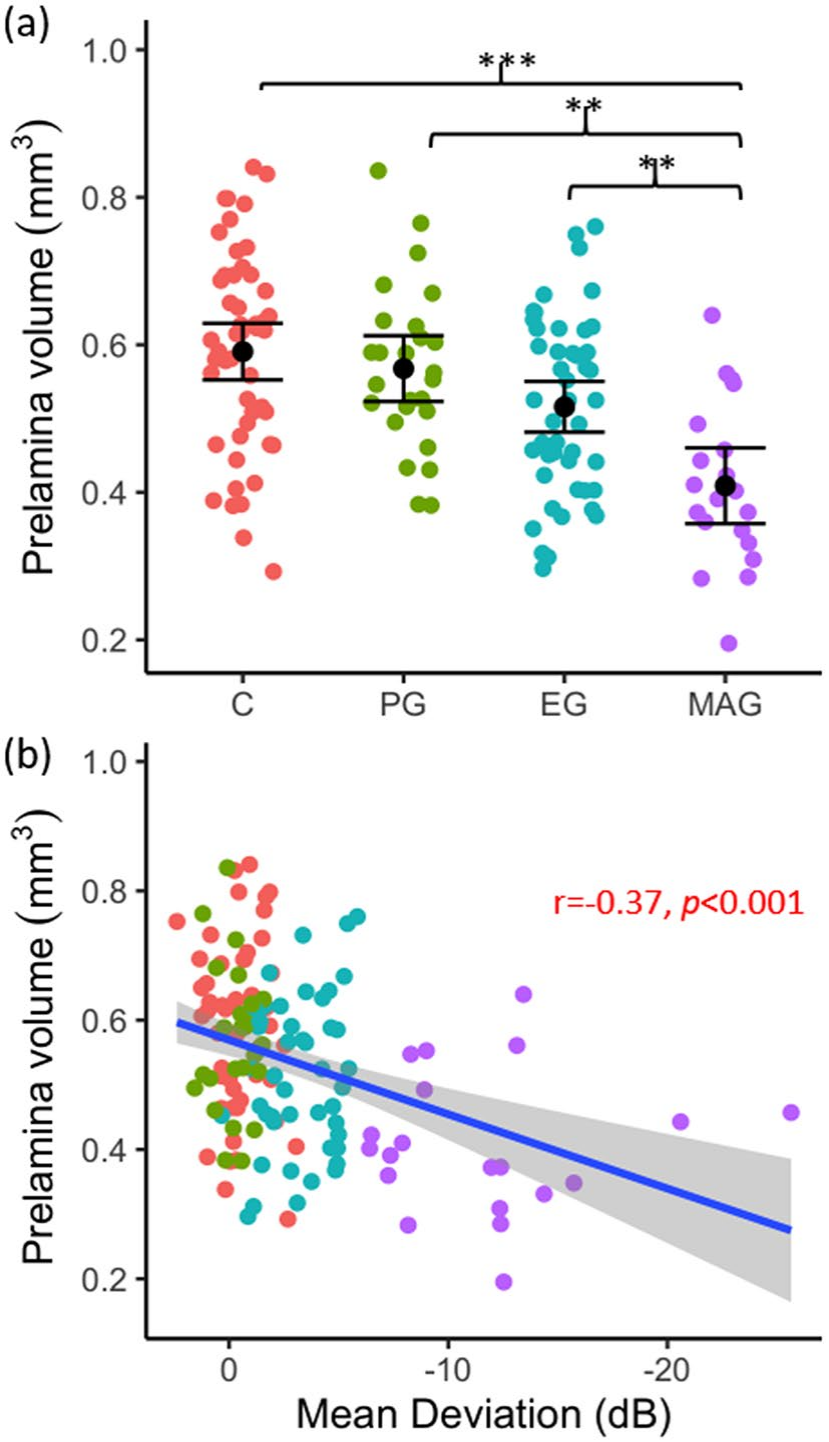
Prelamina volume as a function of glaucoma disease stage (**a**); black point represents mean and error bars indicate 95% confidence intervals, and (**b**) visual field status (VF MD). Blue line represents regression line and grey shading indicates 95% confidence intervals. Red text indicates significant Pearson’s correlation (r) values at *p* < 0.05. *p < 0.05, **p < 0.01, ***p < 0.001.

Prelamina volume (Fig. 7a) did not significantly differ between controls and PG ONHs (*p* = 0.585), or between PG and EG ONHs (*p* = 0.681). However, prelamina volume was significantly less in MAG ONHs than in EG (*p* = 0.007), PG (*p* = 0.001) and control eyes (*p* < 0.001). A significant negative correlation was found between prelamina volume and VF MD (*p* < 0.001, Fig. 7b).

#### Factor-associations with axonal parameters

An association between increasing age and a decrease in mean peripapillary NFL, border NFL, MRW and MRA was determined (*p* < 0.01). Age was negatively associated with border NFL, MRW, and MRA in all regions (*p* < 0.05), except nasal (*p* = 0.245) and inferior nasal (*p* = 0.606) border NFL, nasal (*p* = 0.061) and inferior nasal (*p* = 0.412) MRW, and temporal (*p* = 0.108) and inferior nasal (*p* = 0.279) MRA. Additionally, age was found to have a negative association with prelamina thickness in the inferior and superior nasal regions (*p* < 0.05). Increasing axial length was negatively associated with mean, superior, and inferior MRW (*p* < 0.05), and border NFL in all regions (*p* < 0.05), except nasal (*p* = 0.083), temporal (*p* = 0.088), and inferior temporal (*p* = 0.191). CCT showed a positive association with the superior, superior temporal and inferior nasal prelaminar thickness (*p* < 0.05).

All association factors were accounted for in the relevant statistical models used to analyse the respective axonal parameters, described above, with respect to glaucoma disease stage.

## Discussion

Since vision loss in glaucoma cannot be recovered, early detection and diagnosis is essential. OCT evaluation of RNFL thickness has been increasingly adopted as an objective clinical measure for detection and assessment of glaucoma^34,71^. This study aimed to further investigate in vivo indices of RGC axons that alter in the earliest stages of glaucoma disease or may hold potential to characterise different stages of disease. This is the first study to quantify in vivo measurements of border NFL thickness and prelamina volume, and evaluate regional measures of prelamina thickness as a function of glaucoma disease stage.

The most important finding is that significant differences in border NFL, peripapillary NFL, MRW and prelamina thickness could be seen between control and PG ONHs, demonstrating that alterations in RGC axonal parameters can be quantified and detected in vivo, prior to permanent vision loss. Our finding consistent with previous studies reporting structural damage to the RNFL before clinically detectable loss of vision^24,49,72^. Indeed, Kerrigan-Baumrind et al.^23^ reported a loss of 25% to 35% of RGCs and their axons was associated with abnormalities detected by automated visual field testing.

The prelamina thickness was significantly less in PG ONHs compared with controls in all regions analysed. Average border NFL and peripapillary NFL, superior and inferior peripapillary NFL, as well as inferior and superior nasal MRW significantly decreased in PG ONHs, compared to controls. However, MRA did not significantly differ between PG ONHs and controls in any region analysed. Since border NFL, peripapillary NFL, and MRW were also significantly less in EG ONHs, compared to control ONHs, their potential use in identification of early-stage glaucoma is indicated.

Additionally, various specific regions of border NFL, peripapillary NFL, MRW, and MRA significantly differed between PG, EG, and MAG ONHs, suggesting that other axon-related indices may be useful in determining disease stage. Here, superior temporal peripapillary NFL in PG ONHs was thicker than in EG ONHs, and peripapillary NFL thickness differed between EG and MAG in all regions, except the superior, superior nasal, and inferior nasal regions, consistent with previous studies that reported peripapillary NFL to have good diagnostic sensitivity for glaucoma detection^73–75^, and also indicate disease progression^76,77^. In vivo measurements of peripapillary NFL have been shown to be preferable for glaucoma detection over macula retinal thickness parameters^37,78–80^. Furthermore, Sung et al.^81^ reported that peripapillary NFL thickness (automated measure by Cirrus SD-OCT) outperformed rim area, cup volume, and vertical cup-disc ratio for glaucoma discrimination, particularly in early glaucoma, whilst in advanced glaucoma, rim area and peripapillary NFL were comparable. Similarly, in moderate-advanced glaucoma participants (average VF MD: − 10.4 ± 8.5 dB), Mwanza et al.^33^ reported no difference in glaucoma diagnostic ability between ONH parameters and peripapillary NFL thickness measures acquired using the Cirrus SD-OCT device.

Gardiner et al.^46^ suggested that MRW and MRA may be more sensitive parameters for early glaucoma detection, with peripapillary NFL perhaps preferable for monitoring structural change. However, in this study, MRA did not significantly differ between PG and control eyes in any region analysed. We suggest that parameters such as border NFL, peripapillary NFL, and MRW may be better indicators of early glaucoma onset, whilst border NFL, peripapillary NFL, MRW, and MRA also provide insight into stage of disease.

Our study is the first to quantify specific regions of prelamina thickness and prelamina volume in vivo in human POAG. A significant reduction in regional prelamina thickness and volume was associated with decreasing VF sensitivity. Prelamina thickness was significantly less in PG and EG ONHs compared to controls, although did not significantly differ between PG and EG, or between EG and MAG in any ONH region. This suggests that specific regions of the prelamina thins prior to clinically detectable vision loss. Although no differences in prelamina volume was observed between early glaucoma stages or controls, prelamina volume significantly correlated with VF MD indicating a potential role in clinical monitoring of disease progression. Our findings are consistent with prelamina compression in early stages of glaucoma, followed by neural loss with advancement of glaucomatous optic neuropathy.

Multivariate analyses showed age and axial length to have significant associations with axon-related parameters, and that these factors should be accounted for when applied in a clinical setting for glaucoma assessment. The finding is not surprising in view of the known age-related decline in the density of RGC axons^82^ at a rate of 0.5% per year^83^ and reduced sensitivity across the visual field occurs as part of the normal ageing process^84^. The association of axial length with thinner average, superior, and inferior MRW, and most regions of border NFL could be attributed to larger eyes having larger ONHs^85,86^. MRA adjusts for ocular size and did not show any association with axial length. Additionally, since we report a positive association between CCT and prelaminar thickness in some regions, CCT should also be factored into analyses of OCT-derived measures of prelamina thickness. While prelamina measurements show promise, these measurements can be confounded by the ONH vasculature which results in significant shadowing and OCT signal attenuation.

In summary, here we report on novel quantitative measures of the optic nerve head as a function of different degrees of glaucomatous optic neuropathy as determined by classification of disease stage. Of the nine regions of prelamina thickness measured, all were able to distinguish between ONHs in the preperimetric group or early glaucoma group compared to healthy control optic nerve heads, indicating that prelamina thickness was an important parameter for detection of glaucoma before vision loss was detected. Additionally, the eight specific regions of border NFL were also able to differentiate between those ONHs with early glaucoma and healthy controls, whilst the mean value for border NFL was able to distinguish between successive stages of glaucoma, including between preperimetric glaucomatous optic nerve heads and controls, providing another novel measure for early detection prior to vision loss, and also for monitoring of disease stage. The latter was consistent with the border NFL having a significant correlation with visual field sensitivity, which was stronger than that of prelamina thickness. Prelamina volume, the third novel measure used in this study; was not able to distinguish between control and early-stage glaucoma.

Glaucoma diagnosis remains a challenge due to the slowly progressive nature of the disease and the sensitivity for testing for preperimetric glaucoma (reviewed by^87^. Our data reported here on measurements of specific regions of border NFL and prelamina thickness (previously not reported on) holds potential for a clinical role in prediction of optic nerves at risk of developing glaucomatous optic neuropathy and characterisation of disease stage. Additionally, their structure–function association with visual field sensitivity indicates their use in monitoring of disease stage. The concept of the novel border NFL thickness holds potential for clinical utility as it is derived as a measure from the RPE/Bruch’s membrane opening, considered to be a stable reference plane. The latter is recommended as a robust OCT-based disc margin^88^, thereby making parameters easier to segment (International Nomenclature for OCT panel^89^) easier to segment than traditional circumpapillary RNFL measures, as well as reliable even in tilted discs^90^.

## Data Availability

All authors have full control of all primary data, and they agree to allow Scientific Reports to review their data upon request to the corresponding author.
